# Extreme summer heat and drought lead to early fruit abortion in European beech

**DOI:** 10.1038/s41598-020-62073-0

**Published:** 2020-03-24

**Authors:** Anita Nussbaumer, Katrin Meusburger, Maria Schmitt, Peter Waldner, Regula Gehrig, Matthias Haeni, Andreas Rigling, Ivano Brunner, Anne Thimonier

**Affiliations:** 1 Swiss Federal Institute for Forest, Snow and Landscape Research WSL, Birmensdorf, Switzerland; 2 Swiss Federal Institute of Technology ETH, Zurich, Switzerland; 3 Federal Office of Meteorology and Climatology MeteoSwiss, Zurich-Flughafen, Switzerland

**Keywords:** Forest ecology, Forestry, Fruiting, Drought

## Abstract

Years with high fruit production, known as mast years, are the usual reproduction strategy of European beech. Harsh weather conditions such as frost during flowering can lead to pollination failure in spring. It has been assumed that mast is controlled by flowering, and that after successful pollination, high amounts of fruits and seeds would be produced. However, the extremely hot and dry European summer of 2018 showed that despite successful pollination, beechnuts did not develop or were only abundant in a few forest stands. An in-depth analysis of three forest sites of European beech from the Swiss Long-Term Forest Ecosystem Research Programme over the last 15–19 years revealed for the first time that extreme summer heat and drought can act as an “environmental veto”, leading to early fruit abortion. Within the forest stands in years with fruit abortion, summer mean temperatures were 1.5 °C higher and precipitation sums were 45% lower than the long-term average. Extreme summer heat and drought, together with frost during flowering, are therefore disrupting events of the assumed biennial fruiting cycle in European beech.

## Introduction

For many wind-pollinated forest tree species the occurrence of mast years, i.e. years with abundant synchronised fruit and seed production, is a strategy for generative growth^1–3^. Mast years of European beech (*Fagus sylvatica* L.) are partly controlled by summer weather conditions in the previous two years, as well as by spring weather conditions during the flowering season^4–6^. Several studies from European regions support the theory that key drivers for mast occurrence are cold and wet summers two years before the mast year, warm and dry summers one year before the mast year, and warm and dry springs during the mast year^5–11^.

In a recent study on fruiting intensity of the International Co-operative Programme on Assessment and Monitoring of Air Pollution Effects on Forests (ICP Forests), evidence for a basic biennial mast cycle in European beech could be found^4^. This is in accordance with Matthews^12^, who suggested that European beech inherently follows a biennial mast cycle. He hypothesised that this cycle can be disrupted when environmental conditions are disadvantageous. According to that theory, weather impacts would act as inhibitors for mast years. Recent studies on mast frequency in European beech have indeed revealed that disrupted cycles have commonly occurred in several European regions^4,7,13^. Events such as frosts in spring during the flowering period of European beech prevented pollen formation and subsequently led to pollination failure. The phenomenon of frosts preventing trees from producing fruits has been described as an “environmental veto” and has primarily been observed in oak trees (*Quercus* sp.)^12,14,15^. According to Pearse *et al*.^16^ and Geburek *et al*.^17^, European beech is a species which is controlled by flowering but not by fruit maturation. This means that once pollination is successful, fruits and seeds will most likely be produced.

According to theories on resource dynamics^16,18^, it is expected that resource allocation is impacted by mass fruit production. In earlier studies, European beech showed signs of resource depletion^9,16,18^, which describes the mechanism of reduced biomass production in vegetative parts, in reaction to mast year occurrence^8,19^. On the other hand, the theory suggests that a lack of available resources could lead to fruit abortion after successful pollination, but this has rarely been reported for forest trees^20^ (but see e.g. Goubitz *et al*.^21^). In currently used resource budget models it is assumed that pollen concentration, i.e. flower abundance, needs to reach a certain threshold and then both fruit and seed development is assumed to be successful^15,18,22–24^.

**Figure 1:**
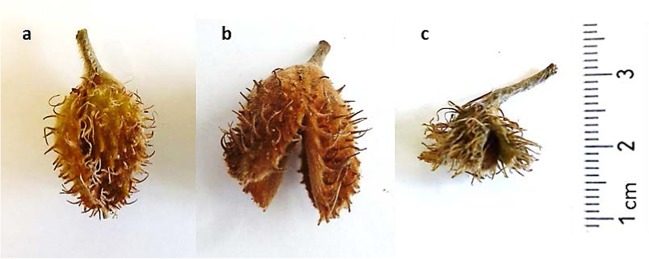
Examples of beechnut development of European beech: (**a**) regularly developing beechnut cupula with beechnuts in summer; (**b**) regularly developed beechnut cupula in autumn after release of beechnuts; (**c**) poorly developed beechnut cupula with beechnuts in summer found in litterfall traps two months before regular beechnut cupulas are typically falling. Photos by Anita Nussbaumer.

In the extremely hot and dry European summer of 2018, beechnuts did not develop properly (Fig. 1), or were only abundant in a few forest stands in Switzerland, despite successful pollination in spring, as indicated by measured high beech pollen concentration (Fig. S1). This led to the assumption that hot and dry summers might affect fruiting behaviour in a similar fashion to frost events. In particular, the summer of 2018 had a prolonged heat and drought period that occurred in many regions of Central Europe and Southern Scandinavia and turned formerly green regions into desiccated and brownish areas^25^ (Buras *et al*., in review).

During the dry and hot summer of 2018 in Switzerland, many forest sites with European beech turned brown already in mid-July and early August, because the trees suffered from early leaf senescence and heavy leaf loss^26^. According to the Swiss Federal Office of Meteorology and Climatology (MeteoSwiss), the whole of Switzerland suffered the strongest summer heat period, and eastern Switzerland suffered also the strongest summer drought since the beginning of measurements in 1864^27,28^. Combined with wind throws due to the heavy storm “Burglind” in the beginning of 2018, this drought and heat stress led to the highest timber harvesting yield in Switzerland since 2011^29^.

In this study, we investigated European beech in three stands, located in the Swiss Plateau, the Jura Mountains and the Prealps (Table S1), providing a unique dataset of diverse external and internal parameters, with data collected over the last 15–19 years. This monitoring takes place in the framework of the Swiss Long-term Forest Ecosystem Research Programme (LWF, ICP Forests Switzerland^30^). We aim at a better understanding of the impact of extreme weather events on mast occurrence and present a novel model for the suggested biennial beech mast cycle including disrupting weather factors. We further investigate environmental triggers for mast years, such as weather cues. Additionally, we investigate the relation between fruit and leaf production, and pollen concentration and leaf production to explore resource dynamics. Beech is a species which shows a distinct leaf growth stop in early summer. In years with high amounts of flower buds, a reduction in leaf biomass might therefore be expected. We analysed pollen concentration, fruit and leaf biomass, as well as soil matric potential, using *t* tests, linear regression models, and generalised linear regression models, to address these issues. We show for the first time that extreme summer heat and drought act as an “environmental veto” for beechnut development despite successful pollination; that both flowering and fruiting in European beech can be triggered by distinct weather patterns; and that abundant flower buds in European beech can lead to decreased leaf production.

## Results

### Impact of drought on fruit development

**Figure 2:**
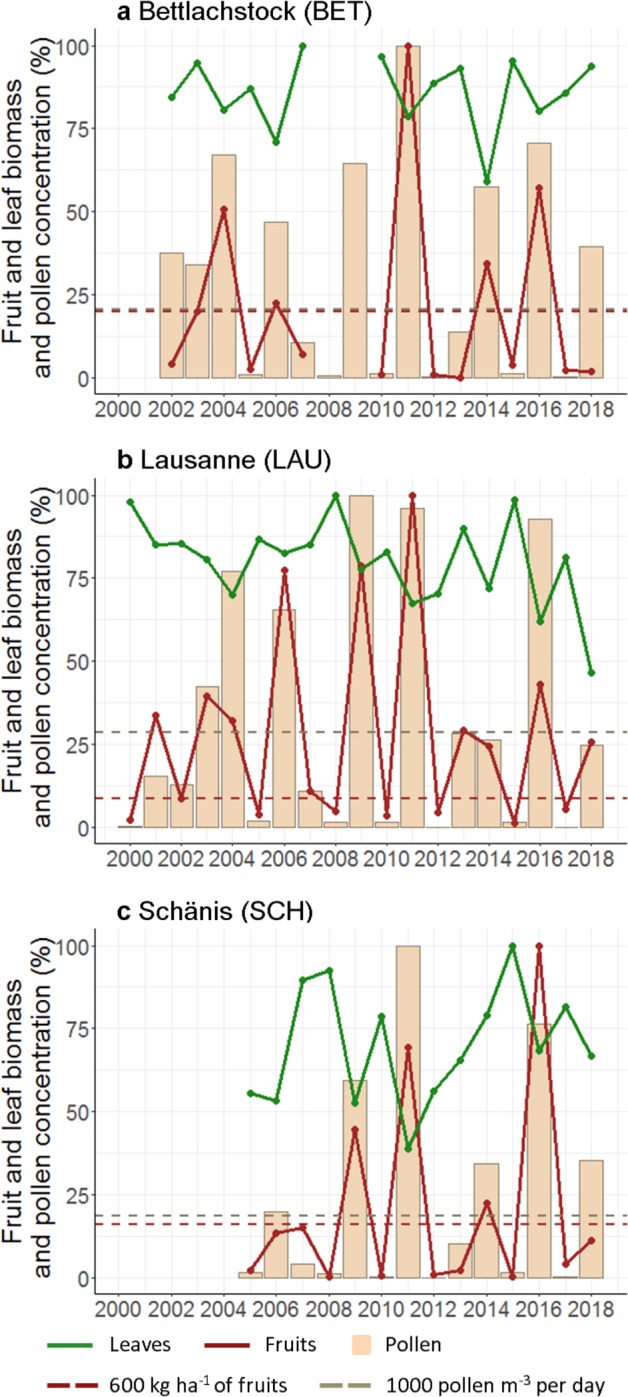
Annual pollen integral APIn (pollen day m^−3^) and leaf and fruit biomass (kg ha^−1^) of European beech in percent of maximum value per site for (**a**) BET, (**b**) LAU and (**c**) SCH. Thresholds for definition of years with fruit abortion: not less than 1000 pollen day m^−3^; no more than 600 kg ha^−1^ of fruits per year. Missing values in BET (2008, 2009) due to lack of litterfall measurements. BET Bettlachstock, LAU Lausanne, SCH Schänis.

**Figure 3:**
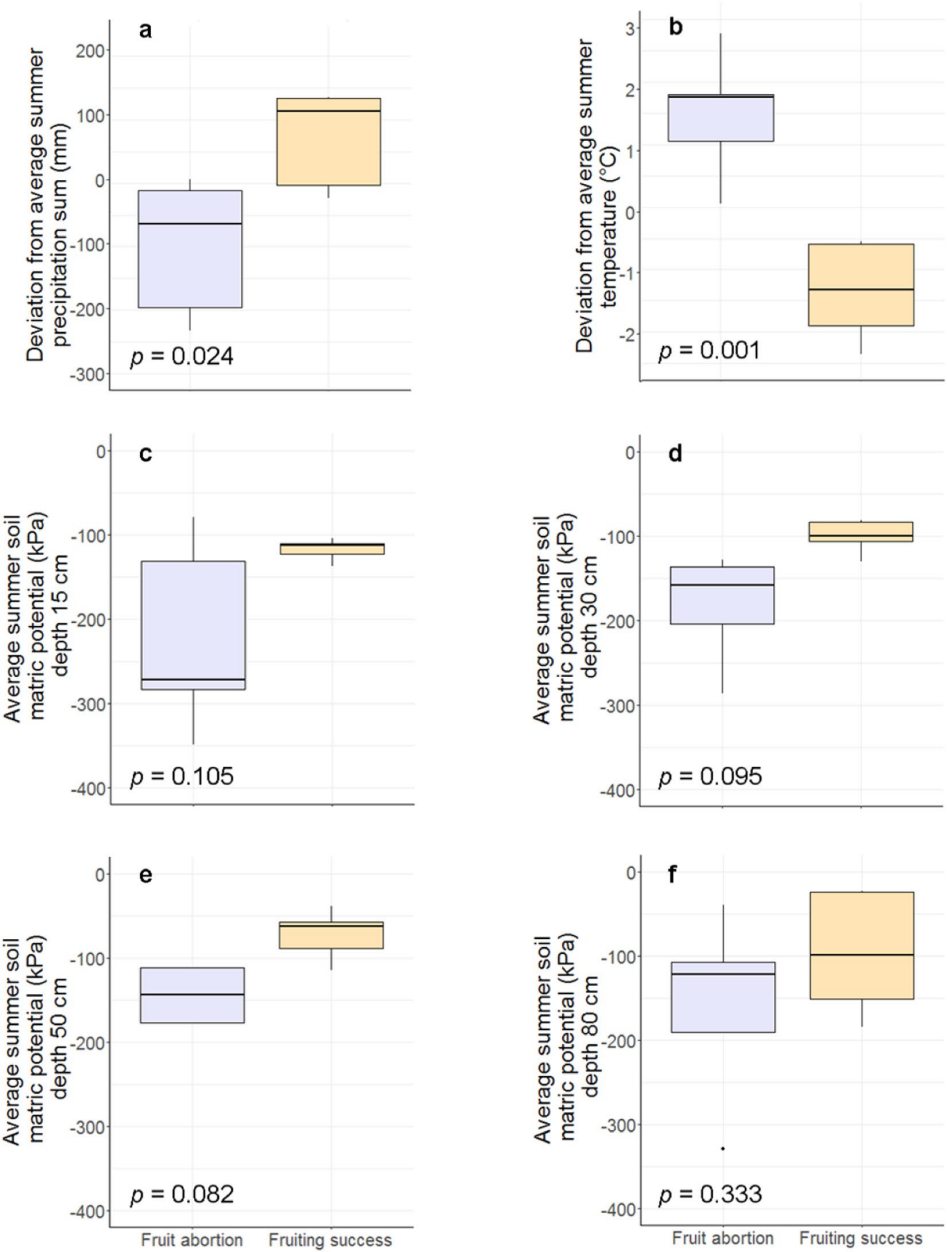
Differences of summer weather conditions and deviations of long-term mean summer soil matric potential between years with fruit abortion and years with fruiting success in European beech inside stands. Deviations from average summer conditions: (**a**) precipitation sums, (**b**) mean temperatures, summer soil matric potential in (**c**) 15 cm, (**d**) 30 cm, (**e**) 50 cm and (**f**) 80 cm soil depth. *p* values from two-sided *t* tests. Years with fruit abortion: BET: 2002, 2003, 2018; SCH: 2006, 2018; years with fruiting success: BET: 2004, 2011, 2014; SCH: 2011, 2016. BET Bettlachstock, SCH Schänis.

European beech fruiting levels from 2006–2018 in Northern Switzerland revealed that fruiting success (mass fruit production) and fruiting failure (low fruit production despite high pollen concentration) occurred several times but differed regionally (Fig. S1). Fruiting failure with fruit abortion at the site Bettlachstock (BET) occurred in 2002, 2003 and 2018 and at the site Schänis (SCH) in 2006 and 2018. In contrast, years with very high fruit production were 2004, 2011 and 2014 in BET, and 2011 and 2016 in SCH (Fig. 2, Table S2). Spring weather conditions, as well as spring soil matric potential measurements, did not differ between years with successful mast and fruit abortion (Table 1). However, comparing summer weather conditions of the ten assigned measurements of fruit abortion and fruiting success, precipitation sums were significantly lower in summers with fruit abortion, with 45% less precipitation than the long-term average (*p* = 0.024, n = 10, two-sided test). At the same time, mean temperatures were significantly higher, with 1.5 °C higher temperatures than the long-term average (*p* = 0.001, n = 10, two-sided test) than in summers with fruiting success (Fig. 3, Table 1). Concerning soil matric potential, there were no differences at *p* < 0.05 between years with fruit abortion and years with fruiting success. However, in summers with fruit abortion, soil matric potential was slightly more negative (low soil moisture) than the long-term average (15 cm: −17 hPa; 30 cm: −44 hPa; 50 cm: −13 hPa; 80 cm: −33 hPa, n = 10, Table 1), and in summers with fruiting success, it was considerably less negative (high soil moisture) than the long-term average (15 cm: +99 hPa; 30 cm: +87 hPa; 50 cm: +109 hPa; 80 cm: +94 hPa, n = 10, Table 1). Potentially, the differences were even higher, but the applied measurement method is limited to −700 hPa. The analysis of the sums or means of both seasons, i.e. spring and summer, showed similar significant differences for mean temperatures only, which were higher in years with fruit abortion (*p* = 0.035, n = 10, two-sided test, Table 1), but precipitation sums and soil matric potential showed no significant difference (precipitation sums: Table 1, soil matric potential: not shown). Results of the weather conditions for the open area measurements were similar to those in the stand and are shown in Table S3.

**Table 1:** *t* test results comparing within-stand weather and soil matric potential conditions between years with fruit abortion and years with fruiting success in European beech.

Parameter	Season	Soil depth	*t* value	df	p value	Mean fruit abortion	Mean fruiting success
Precipitation (mm)	Spring		0.46	7.93	0.659	−5.0	−33.0
Precipitation (mm)	Summer		−2.85	7.26	**0.024**	−99.4	71.0
Precipitation (mm)	Spring and summer		−1.60	7.91	0.150	−104.4	38.0
Temperature (°C)	Spring		0.30	7.99	0.768	0.7	0.3
Temperature (°C)	Summer		4.94	7.58	**0.001**	1.5	−1.3
Temperature (°C)	Spring and summer		2.71	5.96	**0.035**	1.1	−0.5
Soil matric potential (hPa)	Spring	15 cm	−0.73	4.33	0.500	−53.8	−92.8
Soil matric potential (hPa)	Spring	30 cm	−0.61	5.24	0.570	−24.1	−47.1
Soil matric potential (hPa)	Spring	50 cm	−0.79	4.64	0.467	−17.5	−31.7
Soil matric potential (hPa)	Spring	80 cm	−1.51	4.04	0.204	−9.5	−56.2
Soil matric potential (hPa)	Summer	15 cm	2.07	4.11	0.105	−222.6	−117.3
Soil matric potential (hPa)	Summer	30 cm	2.15	4.15	*0.095*	−240.8	−100.1
Soil matric potential (hPa)	Summer	50 cm	2.23	4.48	*0.08*2	−196.0	−72.1
Soil matric potential (hPa)	Summer	80 cm	1.04	6.98	0.333	−157.5	−96.3

Years with fruit abortion: BET: 2002, 2003, 2018; SCH: 2006, 2018; years with fruiting success: BET: 2004, 2011, 2014; SCH: 2011, 2016. *p* values in bold: *p *< 0.05, *p* values in italics: *p* < 0.1, df degrees of freedom, Mean fruit abortion and mean fruiting success: mean deviation in years with fruit abortion and fruiting success, respectively, from long-term means. BET Bettlachstock, SCH Schänis.

### Weather impacts on pollination and mast year occurrence

The analysis of weather impacts which lead to years with high pollen concentration (potential mast years) or fruit biomass (mast years) revealed that both the basic model and the ΔT model showed similar coefficients of determination (pollen concentration: *R*^2^ = 0.47; fruit biomass: *R*^2^ = 0.30, in-stand meteorological measurements, Table 2). For pollen concentration, the best fitting models indicated that years with high pollen concentration occurred after a cold summer two years before the potential mast years, and a warm summer one year before the potential mast years (large positive deviation of summer mean temperatures), and a dry spring during the pollination season (Table 2). The most influential weather impacts on fruit biomass were similar to those for pollen production, but spring precipitation in the mast year was not included in the best fitting models (Table 2). For both tested parameters, pollen concentration and fruit biomass, both models did not substantially differ from each other (pollen concentration: *p* = 0.143; fruit biomass: *p* = 0.652). The analysis of the open area meteorological measurements showed that for pollen concentration, the best fitting weather regression models were similar to the in-stand models, although the coefficients of determination were lower (*R*^2^ = 0.41, Table S4). The best fitting models for fruit biomass differed from the in-stand models and included high summer precipitation sums two years before the mast year and low summer precipitation sums in the year before the mast year. Temperatures were not part of the models, and the coefficients of determination were similar to those of the in-stand weather regression models (*R*^2^ = 0.30, Table S4).

**Table 2:** Best fitting regression models for the impact of weather conditions on pollen concentration and fruit production of European beech stands.

Generative variable	Model	*R*^2^	Difference of summer temperatures 1 and 2 years before target year	2 years before target year (summer)		1 year before target year (summer)		target year (spring)	
				Temperature	Precipitation	Temperature	Precipitation	Temperature	Precipitation
Pollen	Basic	0.47	xxx	↘	0	**↘**	0	0	↘
Fruits	Basic	0.30	xxx	↘	0	**↘**	0	0	0
Pollen	ΔT	0.47	**Δ**	xxx	0	xxx	0	0	↘
Fruits	ΔT	0.30	**Δ**	xxx	0	xxx	0	0	0

Meteorological measurements from stations inside the stands, deviations from long-term mean. Basic model: includes summer (June and July) mean temperatures and precipitation sums of the two years before the target year, and spring (April and May) mean temperatures and precipitation sums of the target year. ΔT model: includes difference between summer (June and July) temperatures of the two years before the target year, summer precipitation sums of the two years before the target year, and spring (April and May) mean temperatures and precipitation sums of the target year. xxx: parameter not part of the model. ↘ = lower than average, **↘** = higher than average, Δ = summer temperature difference relevant, 0 = not included in the best fitting model. *R*^*2*^ from linear regression models.

### Relations between pollen and fruit biomass production

**Figure 4:**
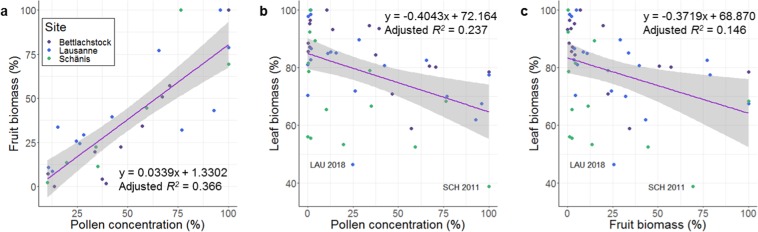
Linear regression models of the compartments fruit biomass, leaf biomass and pollen concentration. (**a**) pollen concentration and fruit biomass, (**b**) leaf biomass and pollen concentration and (**c**) leaf biomass and fruit biomass of European beech; in percentage of maximum value per site. For (**a**) only measurements with more than 10% of pollen concentration were used, for (**b**,**c)** all measurements were used. For (**a**) log-transformed fruit biomass was used. Exceptionally low leaf biomass values occurred in SCH in 2011 and in LAU in 2018. Lines: linear regression lines, grey area: 95% confidence interval. LAU Lausanne, SCH Schänis.

Pollen concentration and fruit biomass at all sites showed a considerable positive relation, even when excluding the lowest 10% of pollen concentration measurements (n = 29, adjusted *R*^2^ = 0.366, *p* < 0.001; Fig. 4, Table S5). At the site scale, a similar positive relation between pollen concentration and fruit biomass was present (BET: n = 10, adjusted *R*^2^ = 0.474, *p* = 0.017; LAU: n = 12, adjusted *R*^2^ = 0.604, *p* = 0.012; SCH: n = 7, adjusted *R*^2^ = 0.730, *p* = 0.009; Fig. S2, Table S5). However, years with pollen concentration between 40 and 99% at all sites showed a weaker positive relation (n = 11, adjusted *R*^2^ = 0.240, *p* = 0.072; Table S5).

### Resource allocation

Leaf biomass was reduced in years with high pollen concentration or fruit biomass at all sites (n = 48, pollen: adjusted *R*^2^ = 0.237, *p* < 0.001; fruit: adjusted *R*^2^ = 0.146, *p* = 0.004; Fig. 4, Table S5). At the site scale, a reduction of leaf biomass in years with high fruit biomass was only present at BET (n = 15, pollen: adjusted *R*^2^ = 0.205, *p* = 0.051; Fig. S2, Table S5). In years with high pollen concentration, however, leaf biomass was reduced at all three sites (BET: n = 15, adjusted *R*^2^ = 0.315, *p* = 0.017; LAU: n = 19, adjusted *R*^2^ = 0.192, *p* = 0.035; SCH: n = 14, adjusted *R*^2^ = 0.271, *p* = 0.033, Fig. S2, Table S5). There was no change in leaf biomass in relation to pollen concentration or fruit biomass of the previous year (Table S5).

## Discussion

Early fruit abortion in European beech has, to our knowledge, not been previously reported. A recent study^14^, however, reported that environmental factors such as frosts can lead to a pollination failure via an “environmental veto”, inhibiting fruit setting, mainly observed in oak trees. Our observations in European beech led to the conclusion that even if pollination in spring, and therefore fruit setting, is successful, subsequent fruit production can fail. In 2018, fruit development was hampered and fruits were aborted in summer, as verified by the litter trap capture (Fig. 1). This happened most likely due to the extended extreme hot and dry weather conditions. Fruit abortion in temperate forests, however, is only reported for a few tree species such as oak (*Quercus alba* L.), hazel (*Corylus maxima* Mill.), and pines (*Pinus spp*.), and only when the fruits have been damaged by late frosts or insects^20,31^. The abortion of intact fruits, in contrast, has been reported mostly for orchard trees, but rarely for forest trees (but see Goubitz *et al*. 2002)^21^. It seems likely to be a response to limited resources, such as leaf reduction due to herbivory, defoliation, or leaf shading^20^. In our case, for European beech, early browning or loss of leaves (“defoliation”) as a consequence of the extremely hot and dry summer likely led to a shortage of resources, although other factors, e.g. genetic expression or hormonal regulation, can play a role as well^32^.

The assumed biennial mast cycle in European beech can be interrupted by environmental factors, as seen in the fruiting levels from 2006 to 2018 (Fig. S1). Such interruptions most likely occur due to spring frosts (“environmental veto”^14^) or long periods of precipitation, both of which can lead to pollination failure. An example of the latter can be seen in 2013 when there were wet (and cold) weather conditions until late May^33^, which most probably prevented successful pollination, and late frost in higher altitudes which likely led to early fruit abortion (as described by Stephenson^20^). This specific weather situation may have led to low fruiting levels for most parts of Switzerland, and only in the northern Swiss Plateau were fruits abundant. Concerning summer weather conditions during years with successful pollination in spring, cool and wet conditions in summer were shown to be favourable for fruit development.

Although dry and hot conditions during the vegetation period are well known to act as an inhibitor to stem growth^34–36^, this effect could not be found at the LWF sites in 2018, as seen by LWF annual stem growth measurements (unpublished data). However, in contrast to fruit abortion, which has a terminal effect on fruit development, stem growth can be continued when soil matric potential increases after a drought period. Soil matric potential was measured within a humidity range which is not expected to affect tree growth. The findings in this study therefore suggest that European beech might react more sensitively to abortion of fruits after successful pollination than to a decrease in vegetative growth. Especially in SCH, precipitation sums in spring and summer of 2018 were the lowest in the 15 years of measurements. This could be evidence that European beech trees from mesic provenances react more sensitively to drier conditions. On the other hand, at the LAU stand, fruit production did not seem to be hampered by hot and dry conditions in the last 19 years. This might be a result of a combination of a more favourable soil type, compared to BET, and a lower dependency of the trees on soil moisture conditions, compared to SCH (Table S1).

An early abandonment of fruit development if conditions are unfavourable is in accordance with the suggestion of Waring^37^ and Dobbertin^38^ that foliage and bud growth are the most important tree compartments, followed by root and stem growth, and fruit development ranks very low. If conditions for fruit production are not favourable, trees stop resource allocation to fruits to lower the risk of resource depletion. They will produce flower buds for the following year instead. A reduction in leaf production in flowering and, to a lesser degree, mast years regularly occurred on our study sites, which is an indication of resource switching during years with potential fruit production (Figs. 4, S2). This is a resource dynamics mechanism describing the assumption of a constant annual resource budget that will be shared between compartments, leading to a shift of resource allocation away from vegetative compartments towards fruits in mast years^2,9,16^. However, the findings in this study strongly suggest that additional defoliation during droughts reduces available resources below the limits needed for fruit development. This could be seen best at the BET site in 2002 to 2004, when 2002 and 2003 showed fruit abortion, and only 2004 was a mast year. In these three years pollen concentration was high in every spring (Fig. 2, Table 1). In 2019, after fruit abortion in 2018, spring weather conditions most probably led to low pollen concentrations. However, during the Sanasilva crown condition survey campaign in summer 2019, sporadic fruiting was present in Swiss European beech stands (Sanasilva programme, unpublished data) which is evidence of a potential flowering year. These findings are in contrast to Geburek *et al*.^17^ who suggested that pollination in European beech only occurs if weather conditions in the previous years were favourable. According to our data, it seems that European beech produces flowers every year until fruit production is successful, and only in the years of a successful mast flower buds are not created. This then results in low pollen concentration in the following spring. Signs of resource depletion therefore only occur after successful fruit production, but not if the fruits were aborted. The negative impact of high fruiting levels on the production of flower buds within the same season is clearly visible for our study region where, after the successful mast years of 2006, 2009, 2011 and 2016, no fruits occurred (Fig. S1). In 2014 however, resource depletion was not observed, which strongly suggests that 2013 was not a successful fruiting year.

**Figure 5:**
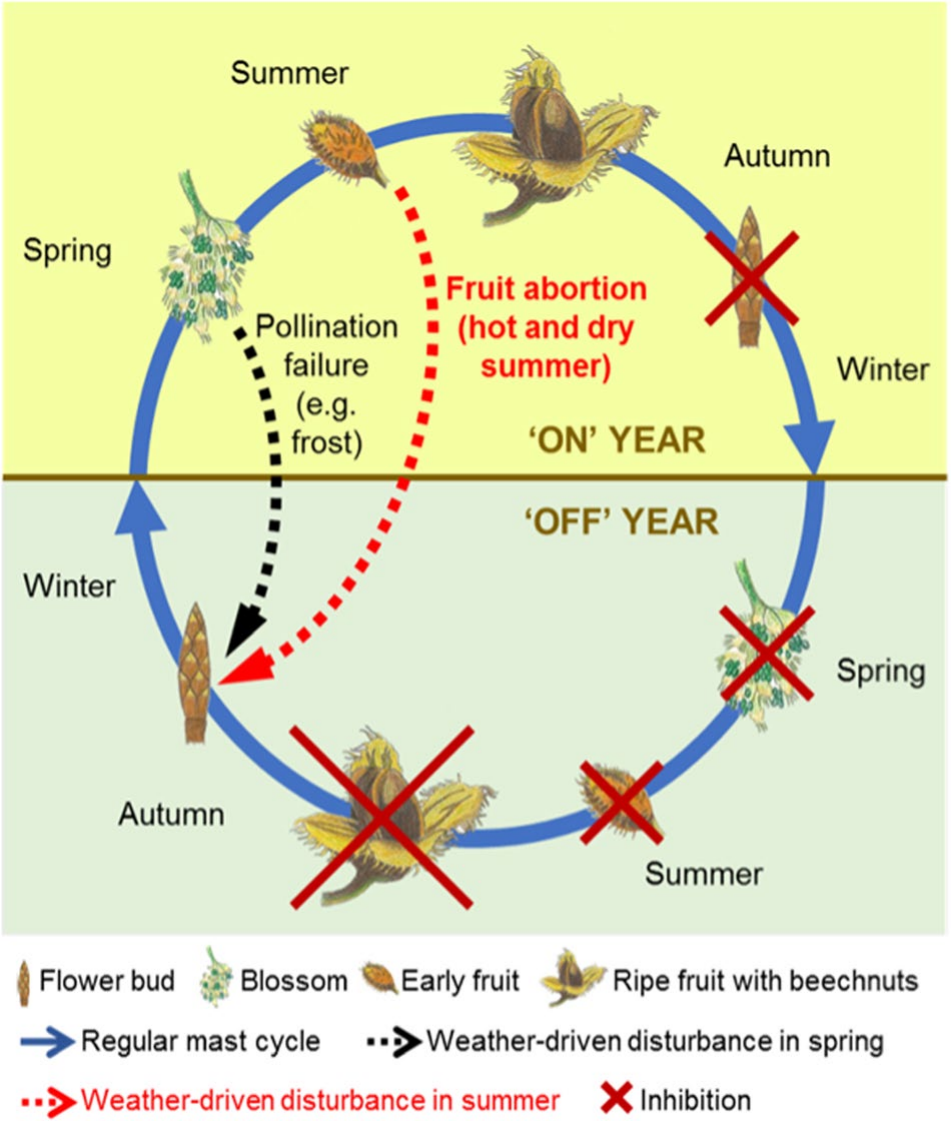
Model of biennial mast cycle in European beech. Basic biennial mast cycle of European beech with weather-driven disturbances in spring leading to pollination failure (dashed black arrow) and, newly postulated, in summer due to extremely hot and dry conditions leading to fruit abortion (red arrow). ‘On’ year = potential mast year, ‘Off’ year = year after successful mast year, without flower buds. Scheme according to Lavee (2007)^40^. Original artwork by Anita Nussbaumer.

The findings in this study support the assumption that European beech has a basic biennial mast cycle, similar to the olive tree (*Olea europaea* L.)^39^, where years with fruits are described as “on” years, and years without fruits as “off” years. The biennial mast cycle of European beech was first hypothesised by Matthews^12^. We show here that these cycles can be interrupted by unfavourable summer weather conditions (Fig. 5). The underlying biennial cycle may be partly influenced by weather conditions during the vegetation period and the summer conditions in previous summers, and partly by resource dynamics of the trees. As aforementioned, other mechanisms, such as gene expression or hormonal regulation might play a role as well^32^. Fruit development for beech seems to be very resource intensive and therefore, trees typically do not develop flower buds for the next year during summers of successful mast years, as seen in the pollen data for Switzerland (Figs. 2 and S1). Therefore, the following year will be a non-mast year, but flower buds will be produced during summer in preparation for a subsequent potential mast year. Overall, we postulate that the biennial mast cycle of European beech can be interrupted by three weather-driven disturbances: i) frosts in spring, ii) long rainy periods in spring, both leading to pollination failure, and iii) extremely hot and dry summers leading to fruit abortion, despite successful pollination in spring.

Observations of weather extremes in Europe show that in the last two decades, heat extremes became more abundant with a general increase of maximum temperatures by 1.5 °C, which is a consequence of recent climate change^40^. According to Swiss climate change scenarios^41^, the climate in the near future will change to hotter and drier summers in Middle Europe, including Switzerland, and therefore, the risk of extended extreme summer heat and drought periods will most probably increase in the next few decades. The RCP 8.8 scenario predicts a temperature increase of 3.3–5.4 °C until 2100 for Switzerland, and at the same time precipitation is predicted to increase in winter, but summers will become drier^41^. Hence, it is likely that European beech will face heavy future challenges and might be replaced by more heat- and drought-tolerating tree species such as oak species^42^. Our study shows that extreme summer heat and drought can lead to mast failure and, as such weather situations will most likely occur more often in the coming decades, natural regeneration for some forest tree species could become a problem in Switzerland and other European regions. We have shed further light on mast cycles in beech and provided evidence for a basic biennial mast year pattern that can be interrupted by unfavourable weather conditions.

## Methods

### Study sites

To investigate the triggers and impacts of mast years in beech, observations from three beech sites of the Swiss Long-Term Forest Ecosystem Research Programme (LWF, ICP Forests Switzerland) were analysed (Fig. S1, Table S1). The site Lausanne (LAU) is situated at the border of the southern part of the Swiss Plateau at 800 m asl. The site Bettlachstock (BET) at 1100–1200 m asl represents conditions in the Jura Mountains. The site Schänis (SCH) is located in the eastern Prealps at 700–770 m asl. At these sites several measurements and assessments are regularly performed to investigate forest ecosystems. The parameters from the LWF which were used in this study derive from litterfall and deposition collection, meteorological measurements and soil matric potential measurements; pollen concentration measurements derive from the national pollen monitoring network^43^ operated by the Federal Office of Meteorology and Climatology MeteoSwiss (www.meteoswiss.ch, personal communication).

### Litter collection

In accordance with the manual of ICP Forests^44^, litterfall is continuously collected in permanent litter traps (10 per stand) with a collection area of 0.25 m^2^, which are emptied every two to eight weeks, depending on seasonal amount of litterfall. After drying, the samples are sorted into different fractions, a mean dry weight (biomass) per stand and collection period is calculated, and annual biomass values are obtained by summing up dry weights over the vegetation year (April to March). In this study the three fractions beechnuts, beechnut cupulas and beech leaves were considered. Up until 2010, beechnuts and cupulas were not sorted separately and were therefore investigated as one parameter.

### Meteorological measurements

Air temperature derives from the meteorological stations in the stands and from the corresponding open field areas nearby^45^, and sums of precipitation are collected in the scope of deposition measurements^46^. Collection methods follow the manuals of ICP Forests (meteorology: Raspe *et al*.^47^ deposition: Clarke *et al*.^48^). Temperature measurements are taken every minute and averaged to 10 minutes (Rotronic MP103A Humidity/Temperature probe). Precipitation sums are continuously collected and aggregated to a bi-weekly sum; inside the stand as throughfall with 16 funnel-type collectors, and in the corresponding open field area as regular precipitation with three similar collectors. During winter at BET and SCH, the funnel-type collectors are replaced with snow buckets (four inside the stand, one in the open field).

### Soil water measurements

Soil matric potential as a measure for plant available water is measured manually with a hand held manometer (Leo 1, from Keller, Switzerland) every two weeks. Tensiometers consist of a plexiglass tube with a round bottom tapered ceramic neck cup (from AgroTerra GmbH, Switzerland), installed in 8 depth replicates at 15, 30, 50 and 80 cm. The measurable range covers water matric potential up to −900 hPa^49^.

### Pollen measurements

Pollen concentration measurements are collected with volumetric pollen traps (Hirst design) by the Federal Office of Meteorology and Climatology MeteoSwiss^50^. The annual pollen integral (APIn) is calculated by summing up the daily average pollen concentration for the year^51^ (Fig. 2). For LAU, pollen measurements of the station Lausanne were used (10 km south of LAU), for SCH, measurements from Buchs SG (30 km east of SCH) were used, and for BET, an average from the measurement stations in Neuchâtel (45 km southwest of BET), La-Chaux-de-Fonds (50 km southwest of BET) and Basel (35 km north of BET) was calculated. Pollen concentration data is considered to be representative for an area of 30–50 km around a pollen trap^50^.

### Geospatial interpolation

A geospatial interpolation of observed fruiting intensity per year from 2006–2018 (Fig. S1) was performed with ArcGIS Desktop (version10.7.1), using Bayesian empirical kriging^52^. This is a well-fitting method for small datasets since it accounts for error in estimating the semivariogram through repeated simulation. Here, we used a power semivariogram and restricted maximum likelihood (REML) for parameter estimation. Swiss European beech data from three sources (see Nussbaumer *et al*.^4^) were used: (a) litterfall data from the LWF sites, continuously measured since 2000 (LAU), 2002 (BET), 2005 (SCH) and 2015 (Lägeren); (b) fruiting intensity of single trees, annually assessed on Sanasilva sites (Swiss ICP Forests Level I plots) since 2006 (n = 20); and (c) fruiting intensity of selected stands, annually assessed by Swiss Federal Institute for Forest, Snow and Landscape Research WSL since 1983 (n = 11). Maximum measured or assessed values per plot were defined as 100 percent. The potential distribution of European beech in the Swiss Plateau, the Prealps and the Jura Mountains was estimated in a species distribution model^53^.

### Statistical methods

The software R was used (version 3.6.2) for the three applied statistical analyses. First, meteorological triggers for years with high pollen concentration but little fruit biomass (=fruiting failure) were investigated by comparing years with fruit abortion with years with most successful fruit production. Thresholds for years with fruit abortion were defined via comparison of all measured years with an APIn of more than 1000 pollen day m^−3^ and less than 600 kg ha^−1^ of fruits, resulting in a total of five years from BET and from SCH. They were matched with the same amount of strongest mast years from these two sites (Table S2). Soil matric potential from BET for the summer 2016 was not available and hence, despite being a mast year, it could not be included in the statistical analysis. Measurements from LAU were not included, as on this site, fruit abortion has not occurred in the last two decades. Deviations from the mean precipitation sums, mean temperatures, and soil matric potential in 15, 30, 50 and 80 cm depth as a proxy for potential drought stress were calculated for the two seasons spring (April and May) and summer (June and July). Two-sided *t* tests were performed (‘t.test’ function from the R package ‘stats’^54^) to compare the deviation from mean seasonal temperatures and precipitation sums, and soil matric potential for spring, summer, and the sums or means of both seasons between years with fruit abortion (n = 5) and fruiting success (n = 5). The *t* tests were calculated separately for weather conditions in stands and open areas. However, the open area models are not discussed in this study, as they were performed solely for comparability to studies without local weather measurements.

Secondly, a generalised linear regression modelling was applied (‘glm’ function from the R package ‘stats’^54^) for the analysis of weather conditions leading to mast years. Since the observed data were zero-inflated, we used the binomial family. Deviations from the mean precipitation sums and mean temperatures were calculated for the two seasons spring (April and May of the recent year) and summer (June and July of the two previous years), in accordance with results from previous studies^5–11^. Kelly *et al*.^55^ found that summer temperature difference between two and one year prior to the mast years is a better predictor for mast occurrence than both summer temperature conditions separately, and therefore, two model types were tested, one with deviations of mean summer temperatures of the two previous summers (basic model), and one with the temperature difference between the first and the second year prior to mast (ΔT model). Regression models for the basic (1) and the ΔT model (2) were performed:

1 $${\rm{y}}={\rm{\beta }}0+{\rm{\beta }}1\ast {\rm{t}}0+{\rm{\beta }}2\ast {\rm{p}}0+{\rm{\beta }}3\ast {\rm{t}}1+{\rm{\beta }}4\ast {\rm{p}}1+{\rm{\beta }}5\ast {\rm{t}}2+{\rm{\beta }}6\ast {\rm{p}}2\,+{\rm{\varepsilon }}$$

and

2 $${\rm{y}}={\rm{\beta }}0+{\rm{\beta }}1\ast {\rm{t}}0+{\rm{\beta }}2\ast {\rm{p}}0+{\rm{\beta }}3\ast {\rm{p}}1+{\rm{\beta }}4\ast {\rm{p}}2+{\rm{\beta }}5\ast ({\rm{t}}1-{\rm{t}}2)+{\rm{\varepsilon }}$$

where y is either pollen concentration or fruit biomass, t0 and p0 are deviations from spring mean temperatures and precipitation sums of the investigated year, t1 and p1 are deviations from summer mean temperatures and precipitation sums of the previous year, t2 and p2 are deviations from summer mean temperatures and precipitation sums of the penultimate year, and ε is the error term. The weather predictors were tested for multicollinearity (variance inflation factor <4, ‘vif’ function from the R package ‘car’^56^). The best fitting models were chosen by comparing the corrected Akaike information criterion^57^ (‘dredge’ function from the R package ‘MuMIn’^58^). To test which of the two models (basic model versus ΔT model) fitted best for pollen concentration and fruit biomass, we compared them via an asymptotic likelihood ratio test (‘lrtest’ function from the R package,lmtest’^59^). The models were calculated separately for weather conditions in stands and open areas. However, the open area models are not discussed in this study, as they were performed solely for comparability to studies without local weather measurements.

Finally, for the investigation of the relation between pollen and fruit biomass production, as well as resource dynamics, we used three linear regression models^54^. Tests for normal distribution of the dependent variables were performed (‘shapiro.test’ from the R package ‘stats’^54^). Pollen and litterfall biomass measurements were normalised into the percentage of the maximum of all measured years for each stand. For the analysis of the correlation between pollen concentration and leaf biomass, and fruit and leaf biomass in the same year as well as in the subsequent year, all measured years from the three sites were used. For the investigation of the relations between pollen concentration and fruit biomass, however, values below 10% of pollen concentration per site were excluded. Very low values distort the statistical analyses as no fruit biomass would be expected in years with deficient pollen production. In this linear regression model, fruit biomass was log-transformed for normal distribution.

## Supplementary information

Supplementary information.

## Data Availability

The data that support the findings of this study are available from the corresponding author (A.N.) upon request. Pollen data used in this study are available from the Federal Office of Meteorology and Climatology MeteoSwiss (www.meteoswiss.ch) upon request.
