# Supplementary material for: Extreme summer heat and drought lead to early fruit abortion in European beech
Source: Sci Rep. 2020 Mar 24;10:5334. doi: 10.1038/s41598-020-62073-0 (PMC7093476; doi:10.1038/s41598-020-62073-0)
Supplement: Supplementary file 1 — Supplementary information. [file 41598_2020_62073_MOESM1_ESM.pdf]

SUPPLEMENTARY INFORMATIONS

Extreme summer heat and drought lead to early fruit abortion in European beech

Authors:

Anita Nussbaumer[1,2], Katrin Meusburger[1], Maria Schmitt[1], Peter Waldner[1], Regula Gehrig[3], Matthias Haeni[1], Andreas Rigling[1,2], Ivano Brunner[1], Anne Thimonier[1]

Affiliations:

1 Swiss Federal Institute for Forest, Snow and Landscape Research WSL, Birmensdorf, Switzerland

2 Swiss Federal Institute of Technology ETH, Zurich, Switzerland

3 Federal Office of Meteorology and Climatology MeteoSwiss, Zurich-Flughafen, Switzerland

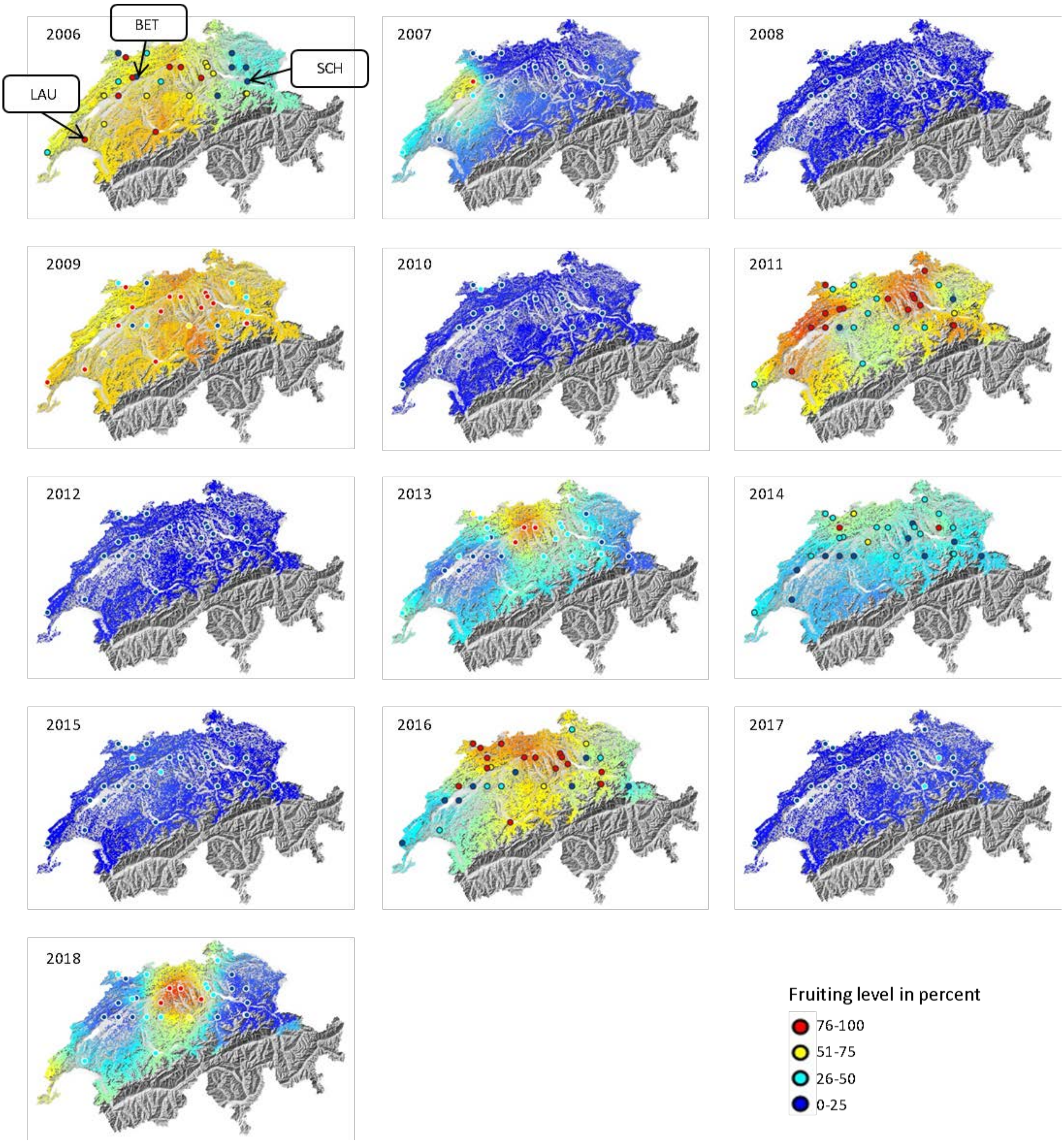

Figure-S1 European beech fruiting levels from 2006-2018 for the Swiss Plateau, the Prealps, Northern Alps and the Jura Mountains, based on the potential distribution of European beech (Wüest et al.[1]). Measurements on stand scale derive from the Sanasilva programme, the LWF and further stand information (Nussbaumer et al.[2]). BET Bettlachstock, LAU Lausanne, SCH Schänis.

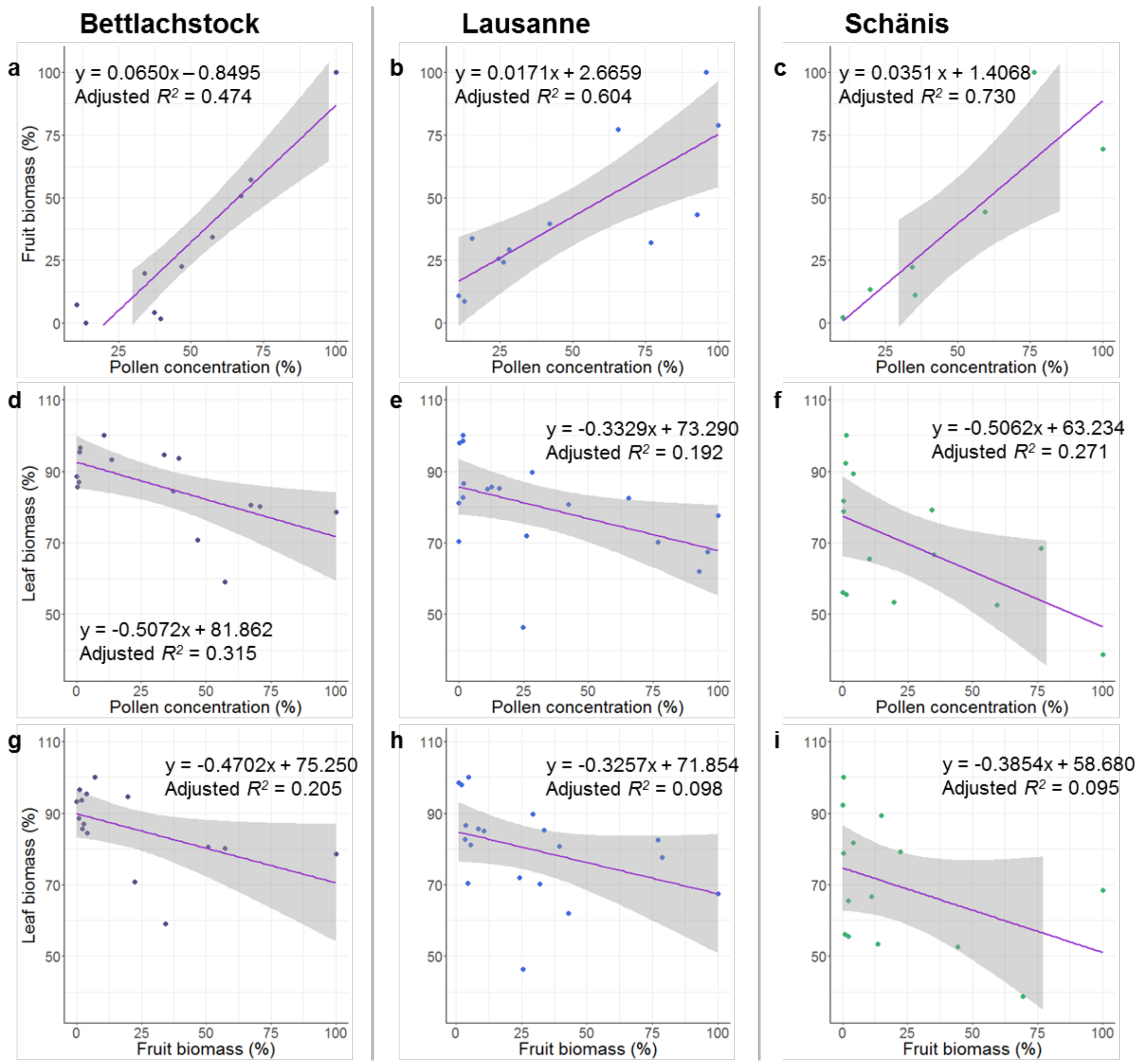

Figure-S2 Linear regression models of the compartments fruit and leaf biomass, and pollen concentration. Linear regression models of pollen concentration and fruit biomass (**a**, **b**, **c**), leaf biomass and pollen concentration (**d**, **e**, **f**) and leaf biomass and fruit biomass (**g**, **h**, **i**) per site: BET: **a**, **d**, **g**; LAU: **b**, **e**, **h**; SCH: **c**, **f**, **i**. All values in percentage of maximum value per site. For **a** to **c** only measurements with more than 10% of pollen concentration were used, for **d** to **i** all measurements were used. For **a**, **d** and **g**, log-transformed fruit biomass was used. Lines: linear regression lines, grey area: 95% confidence interval. BET Bettlachstock, LAU Lausanne, SCH Schänis.

Table-S1 Site characteristics for the three investigated sites.

	**Bettlachstock (BET)**	**Lausanne (LAU)**	**Schänis (SCH)**
Forest type	*Cardamino-Fagetum tilietosum*[a]	*Milio-Fagetum*[a]	*Cardamino-Fagetum tilietosum*[a]
Main tree species	European beech, Silver fir	European beech	European beech
Soil type	Rendzic Leptosol	Dystric Cambisol	Eutric Cambisol
Altitude	1100-1200 m	800 m	700-770 m
Region	Jura	Swiss plateau	Prealps
Annual mean temperature[b]	6.7 °C	7.8 °C	8.8 °C
Annual mean precipitation[b]	1180 mm	1120 mm	1820 mm
Summer mean temperature[c]	14.3 °C	15.5 °C	16.6 °C
Summer mean precipitation[c]	222 mm	197 mm	433 mm
Litterfall data range	2002-2007, 2010-2018[d]	2000-2018	2005-2018

[a] according to Ellenberg and Klötzli[3].
[b] according to LWF data[4, 5]
[c] Average of June, July and August.
[d] incomplete litterfall collection in 2008 and 2009.

Table-S2 Weather conditions, pollen concentration and fruit biomass in years with fruit abortion and years with most successful mast years on the BET and SCH sites. BET Bettlachstock, SCH Schänis.

			Precipitation sum stand (mm)		Precipitation sum open area (mm)		Mean temperature stand (°C)		Mean temperature open area (°C)		Pollen APIn (pollen day m^{-3})	Fruits (kg ha^{-1})
**Mast status**	**Site**	**Year**	**Spring**	**Summer**	**Spring**	**Summer**	**Spring**	**Summer**	**Spring**	**Summer**	**Spring**	**Autumn**
Fruit abortion	**BET**	2002	172.1	181.8	193.2	229.2	7.0	14.4	7.8	15.8	1812	123
Fruit abortion	**BET**	2003	117.8	110.7	147.5	133.0	9.2	17.1	9.0	18.6	1641	597
Fruit abortion	**BET**	2018	118.8	163.1	128.5	224.9	10.6	15.4	11.5	16.9	1912	53
Fruit abortion	**SCH**	2006	420.8	139.7	478.3	194.5	9.3	18.4	10.6	19.9	1062	500
Fruit abortion	**SCH**	2018	160.8	102.6	197.9	140.1	13.1	18.4	13.0	21.8	1890	419
Fruit abortion	**Average**		**198.1**	**139.6**	**229.1**	**184.3**	**9.8**	**16.7**	**10.4**	**18.6**	**1663**	**339**
Fruiting success	**BET**	2004	129.1	171.9	135.2	214.9	6.7	13.0	7.5	14.3	3254	1534
Fruiting success	**BET**	2011	41.4	150.8	61.0	203.2	10.6	12.0	11.2	12.9	4845	3022
Fruiting success	**BET**	2014	154.2	288.1	210.4	369.5	7.8	13.7	8.5	15.1	3428	1039
Fruiting success	**SCH**	2011	158.4	469.4	201.0	559.7	12.8	14.7	13.7	15.8	5372	2582
Fruiting success	**SCH**	2016	367.2	469.9	449.3	597.2	9.7	16.1	11.0	17.7	4101	3720
Fruiting success	**Average**		**170.0**	**310.0**	**211.4**	**388.9**	**9.5**	**13.9**	**10.4**	**15.2**	**4200**	**2379**

Table-S3 *t* test results comparing weather conditions in open area between years with fruit abortion and years with fruiting success in European beech. Years with fruit abortion: BET: 2002, 2003, 2018; SCH: 2006, 2018; years with fruiting success: BET: 2004, 2011, 2014; SCH: 2011, 2016. *p* values in bold: $p < 0.05$, df degrees of freedom, BET Bettlachstock, SCH Schänis.

Parameter	Season	*t* value	df	*p* value	Mean fruit abortion	Mean fruiting success
Precipitation (mm)	Spring	0.26	8.00	0.803	-13.0	-30.6
Precipitation (mm)	Summer	-2.80	6.83	**0.027**	-122.0	82.6
Precipitation (mm)	Spring and summer	-1.73	7.89	0.123	-135.0	52.0
Temperature (°C)	Spring	-0.03	8.00	0.980	0.4	0.4
Temperature (°C)	Summer	4.44	7.49	**0.003**	2.1	-1.3
Temperature (°C)	Spring and summer	3.00	5.13	**0.030**	1.2	-0.5

Table-S4 Best fitting regression models for the impact of weather conditions on pollen concentration and fruit production of European beech stands. Meteorological measurements from open area stations, deviations from long-term mean. Basic model: includes summer (June and July) mean temperatures and precipitation sums of the two years before the target year, and spring (April and May) mean temperatures and precipitation sums of the target year. ΔT model: includes difference between summer (June and July) temperatures of the two years before the target year, summer precipitation sums of the two years before the target year, and spring (April and May) mean temperatures and precipitation sums of the target year. xxx: parameter not part of the model. ↘ = lower than average, ↗ = higher than average, Δ = summer temperature difference relevant, 0 = not included in the best fitting model. R^2 from linear regression models.

Generative variable	Model	R^2	Difference of summer temperatures 1 and 2 years before target year	2 years before target year (summer)		1 year before target year (summer)		target year (spring)	
				Temperature	Precipitation	Temperature	Precipitation	Temperature	Precipitation
Pollen	Basic	0.41	xxx	↘	0	↗	0	0	↘
Fruits	Basic	0.30	xxx	0	↗	0	↘	0	0
Pollen	ΔT	0.41	**Δ**	xxx	0	xxx	0	0	0
Fruits	ΔT	0.30	0	xxx	↗	xxx	↘	0	0

Table-S5 Relations between pollen and litterfall measurements at Swiss and site scale, calculated with linear regression models. In models where fruit biomass was the dependent variable, it was log-transformed. Leaves next year = leaf production in the subsequent year, % = percentage of pollen concentration, all = measurements of all three sites.

Dependent variable	Predictor	Data range	Measured years	Adjusted R^2	*p* value	Relation
Fruits	Pollen	Bettlachstock	10	0.474	**0.017**	positive
Leaves	Pollen	Bettlachstock	15	0.315	**0.017**	negative
Leaves	Fruits	Bettlachstock	15	0.205	0.051	negative
Leaves next year	Pollen	Bettlachstock	14	0.084	0.165	none
Leaves next year	Fruits	Bettlachstock	14	-0.020	0.401	none
Fruits	Pollen	Lausanne	12	0.604	**0.012**	positive
Leaves	Pollen	Lausanne	19	0.192	**0.035**	negative
Leaves	Fruits	Lausanne	19	0.098	0.103	negative
Leaves next year	Pollen	Lausanne	18	-0.049	0.657	none
Leaves next year	Fruits	Lausanne	18	-0.059	0.814	none
Fruits	Pollen	Schänis	7	0.730	**0.009**	positive
Leaves	Pollen	Schänis	14	0.271	**0.033**	negative
Leaves	Fruits	Schänis	14	0.095	0.151	none
Leaves next year	Pollen	Schänis	13	-0.058	0.572	none
Leaves next year	Fruits	Schänis	13	-0.035	0457	none
Fruits	Pollen	All, 10-100%	29	0.366	**<0.001**	positive
Fruits	Pollen	All, 40-99%	11	0.240	0.072	positive
Leaves	Pollen	All	48	0.237	**<0.001**	negative
Leaves	Fruits	All	48	0.146	**0.004**	negative
Leaves next year	Pollen	All	44	0.033	0.121	none
Leaves next year	Fruits	All	44	0.009	0.246	none

References

1. Wüest, R. O., Bergamini, A., Bollmann, K. & Baltensweiler, A. LiDAR data as a proxy for light availability improve distribution modelling of woody species. *For. Ecol. Manag.* **456**, 117644 (2019). 10.1016/j.foreco.2019.117644
2. Nussbaumer, A. et al. Patterns of mast fruiting of common beech, sessile and common oak, Norway spruce and Scots pine in Central and Northern Europe. *For. Ecol. Manag.* **363**, 237-251 (2016).
3. Ellenberg, H. & Klötzli, F. Waldgesellschaften und Waldstandorte der Schweiz. Mitt. Schweiz. Anst. Forstl. Vers.w. 48, 589-930 (1972).
4. Rebetez, M. et al. Meteorological data series from Swiss long-term forest ecosystem research plots since 1997. *Ann. For. Sci.* **75**, 41-48 (2018).
5. Thimonier, A. et al. Total deposition of nitrogen in Swiss forests: comparison of assessment methods and evaluation of changes over two decades. *Atmos. Environ.* **198**, 335-350 (2019).
